# Catecholamine-Induced Reversible Cerebral Vasoconstriction Syndrome: An Overlooked Cause of Thunderclap Headache

**DOI:** 10.7759/cureus.99046

**Published:** 2025-12-12

**Authors:** Kiserah Philip, Andrew Ashworth, Ross Paton, Frances McGrane

**Affiliations:** 1 Acute Internal Medicine, Glasgow Royal Infirmary, Glasgow, GBR; 2 Stroke Medicine, Glasgow Royal Infirmary, Glasgow, GBR

**Keywords:** adrenal mass, catecholamine excess, pheochromocytoma, reversible cerebral vasoconstriction syndrome, secondary headache, thunderclap headache

## Abstract

Reversible cerebral vasoconstriction syndrome (RCVS) is a rare but important cause of thunderclap headache. It is often under-recognized, as the presentation can be variable, and early imaging can be normal. RCVS may be precipitated by catecholaminergic surges, including those from pheochromocytoma.

We describe a 35-year-old female patient who presented with recurrent thunderclap headaches, later complicated by acute confusion and transient dysphasia. Initial investigations were unremarkable, but subsequent magnetic resonance imaging (MRI) revealed a transient parieto-occipital lesion and vascular irregularities, prompting consideration of RCVS. Her evolving clinical picture eventually led to the discovery of a large adrenal mass, which was confirmed via biochemical testing as a catecholamine-secreting pheochromocytoma. Surgical resection following appropriate alpha- and beta-blockade resulted in complete symptom resolution and biochemical normalization.

This case highlights the diagnostic complexity of RCVS secondary to pheochromocytoma, where the absence of typical imaging features and systemic symptoms delayed diagnosis. Early involvement of the multidisciplinary team can be useful in unraveling complex presentations associated with rare secondary causes of headaches. Pheochromocytoma should be considered a potential trigger for RCVS, particularly in patients with unexplained recurrent headaches and systemic symptoms, as early diagnosis and treatment can lead to symptom resolution and prevent serious complications.

## Introduction

Headaches remain a diagnostic challenge in the acute medical setting, with thunderclap headaches representing a particularly urgent presentation. Defined by the International Classification of Headache Disorders (ICHD) as a sudden, severe headache that reaches maximum intensity within one minute and lasts for five minutes or more [1], thunderclap headaches often signal an underlying secondary cause that must be thoroughly investigated.

Reversible cerebral vasoconstriction syndrome (RCVS) is an underrecognized but important cause of recurrent thunderclap headaches, typically resulting from transient cerebral vasoconstriction [1-3]. Although RCVS is generally self-limiting and benign, resolving spontaneously within approximately three months, it can lead to serious complications, including stroke, posterior reversible encephalopathy syndrome (PRES), and intracerebral hemorrhage, if left undiagnosed and untreated [1]. Characteristic angiographic findings of RCVS include a “string of beads” or “sausage on a string” appearance, though early imaging may be normal [1,2].

There is increasing evidence that suggests RCVS can arise from different mechanisms, which can cause transient dysregulation of cerebrovascular tone. This can be triggered by sympathetic overactivity, endothelial dysfunction, and oxidative stress of the cerebral vasculature [4]. Increased sympathetic tone may occur in the peripartum period or be associated with the use of alpha-sympathomimetic agents, serotonergic or illicit drugs, or underlying neoplasms [1,2]. Among the latter, pheochromocytomas, a rare group of catecholamine-secreting neuroendocrine tumors arising from adrenomedullary chromaffin cells [5], represent a rare but critical differential diagnosis. These tumors can precipitate RCVS through episodic catecholamine surges, and patients may initially present with neurological symptoms, complicating the diagnostic process.

We present a case highlighting the complex diagnostic journey of a patient with RCVS secondary to a pheochromocytoma, requiring multidisciplinary collaboration across stroke, cardiology, and endocrinology teams.

## Case presentation

A 35-year-old Caucasian female, with a background of asthma and anxiety, initially presented to the Acute Medical Assessment Unit in January 2023 with a one-week history of debilitating, intermittent, throbbing headache associated with photophobia, nausea, neck pain, and visual distortion. No lacrimation was reported. The episodes lasted up to six hours and were not relieved by paracetamol, codeine, or sumatriptan.

On examination, her blood pressure was 155/53 mmHg, and her heart rate was 65 beats per minute. Neurological examination was unremarkable. She was admitted for further evaluation, where a computed tomography (CT) brain scan and lumbar puncture, performed to exclude subarachnoid hemorrhage, were both unremarkable. She was managed for a suspected atypical migraine or possible cluster headache and discharged with ibuprofen and indomethacin, along with safety-netting advice.

**Figure 1:**
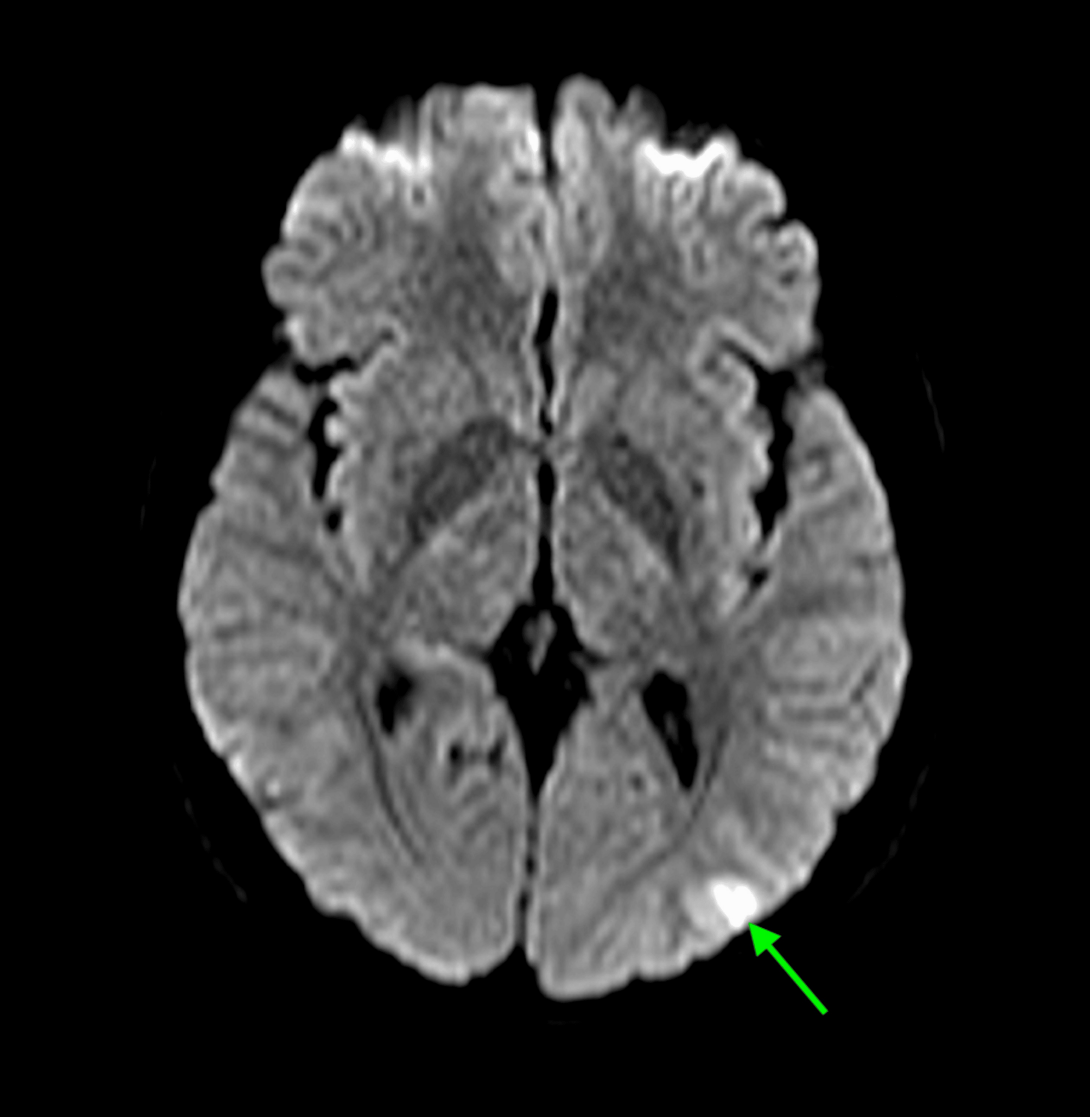
Diffusion weighted MRI of the brain Initial axial diffusion-weighted MRI of the brain demonstrating a hyperintense lesion in the left parieto-occipital cortex (green arrow), initially suggestive of acute infarction or seizure-related changes.

**Figure 2:**
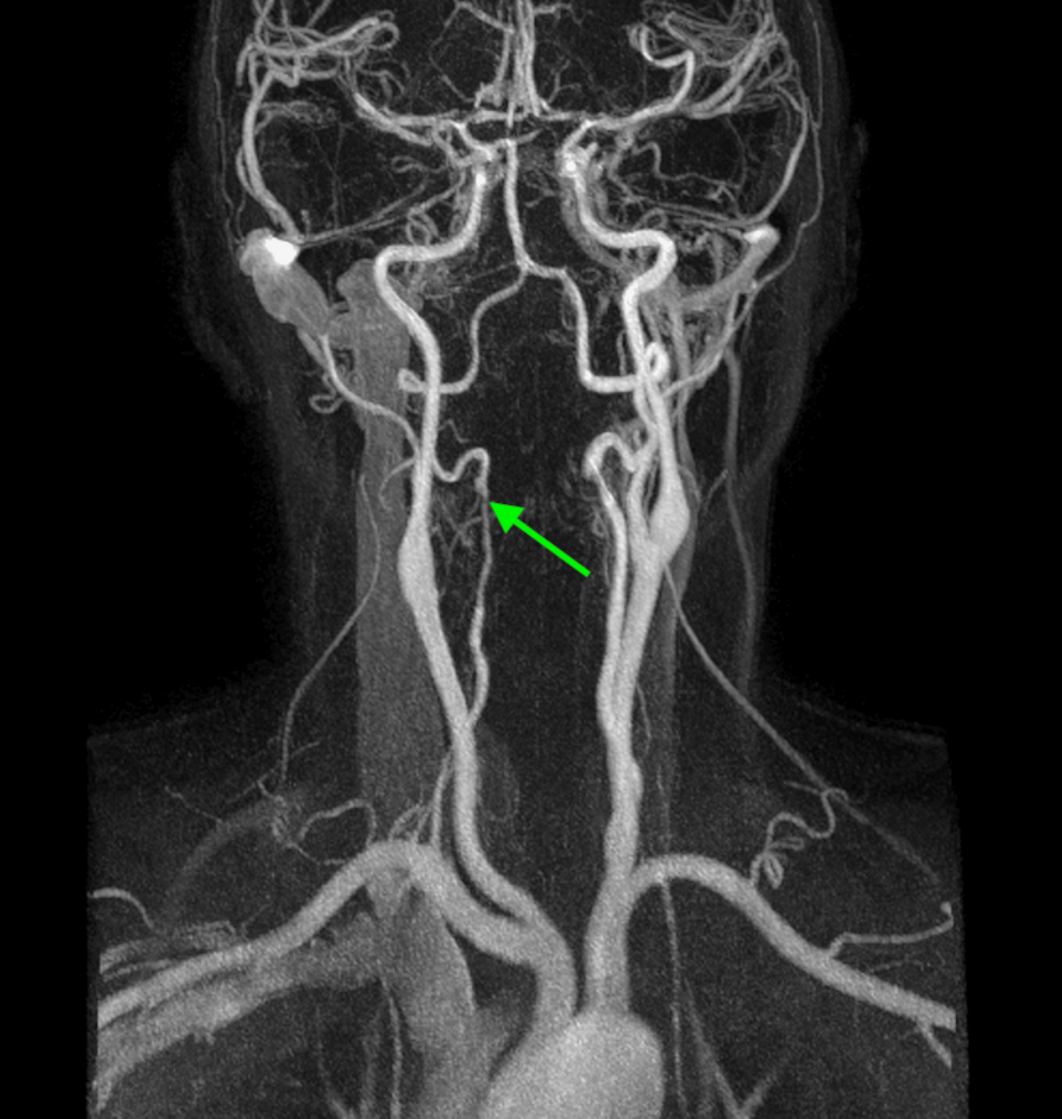
Initial magnetic resonance angiography (MRA) of the head showing irregular flow within the right vertebral artery (green arrow), consistent with segmental vasoconstriction.

In February 2023, she presented again with a frontal headache, acute confusion, and expressive and receptive dysphasia. She had no other focal neurological deficits, and her blood pressure on admission was 146/112 mm Hg, and her heart rate was 72 beats per minute. A repeat CT brain scan remained normal. Given her acute neurocognitive symptoms, she was started on clopidogrel as secondary stroke prevention. A subsequent MRI of the brain revealed a high signal on diffusion-weighted imaging (DWI) in the left parieto-occipital cortex, raising suspicion for a small infarct or seizure-related change (Figure 1). Magnetic resonance angiography (MRA) demonstrated attenuated flow in the mid portion of the right vertebral artery (V2 segment), with reconstitution distally (Figure 2).

**Figure 3:**
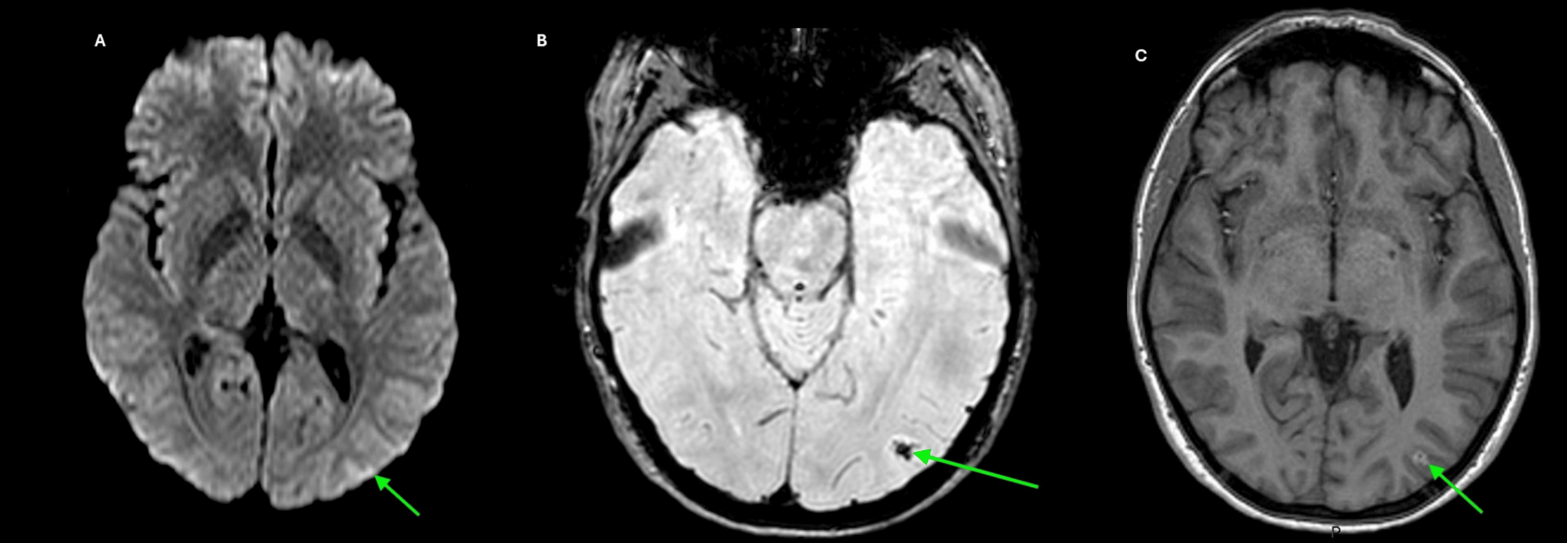
A follow-up brain MRI performed two weeks after the initial scan showing (A) near-complete resolution of the previously identified hyperintense lesion in the left parieto-occipital cortex (green arrow) on the diffusion-weighted sequence, (B) a susceptibility artifact in the left parieto-occipital cortex (green arrow), indicative of hemosiderin deposition and suggestive of prior hemorrhage on susceptibility-weighted imaging (SWI), and (C) cortical enhancement in the left parieto-occipital region (green arrow), supporting the presence of subacute hemorrhage on the post-contrast T1-weighted 3D fast field echo (FFE) MRI sequence.

**Figure 4:**
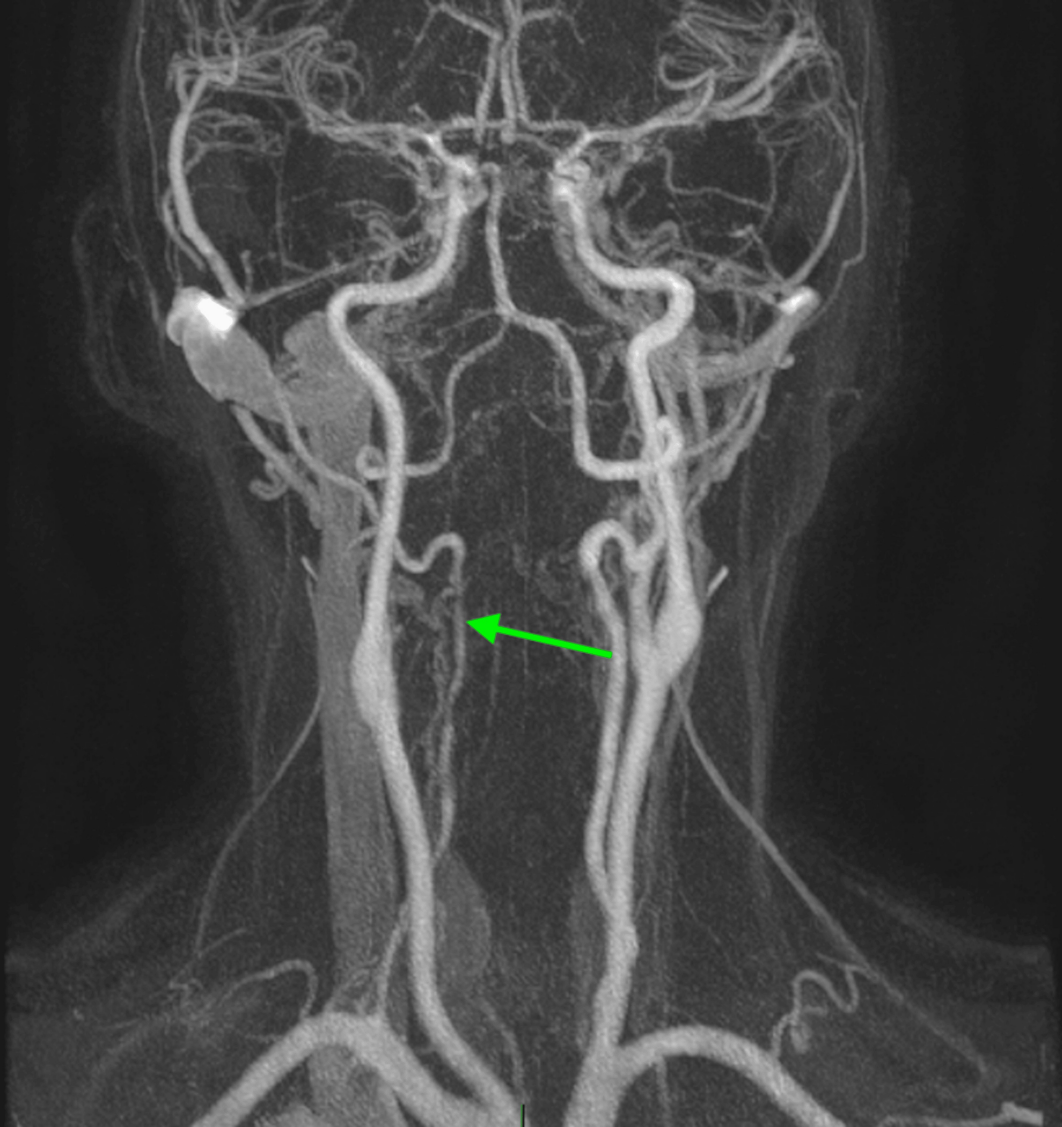
Follow-up magnetic resonance angiography (MRA) of the head at two weeks demonstrating persistent but improved irregular flow in the right vertebral artery (green arrow), remaining consistent with resolving vasospasm.

Subsequent imaging two weeks later showed resolution of the parieto-occipital DWI signal (Figure 3a) but a persistent high signal on T2-weighted fluid-attenuated inversion recovery (FLAIR) and susceptibility artifact on minimum intensity projection (minIP) (Figure 3b), with post-contrast cortical enhancement on T1-weighted 3D fast field echo (FFE) (Figure 3c), indicating focal hemorrhage and blood-brain barrier disruption. MRA continued to show irregularity in the right vertebral artery (Figure 4). A multidisciplinary stroke team discussion favored a small cortical infarct, possibly embolic in origin via the left common carotid system. Differential diagnoses included fibromuscular dysplasia or segmental arterial mediolysis.

At the six-month follow-up, MRI demonstrated persistence of the susceptibility artifact in the left parieto-occipital region. During review by the specialty stroke team, the patient reported ongoing episodes of palpitations, throbbing head and neck pain, and vomiting, lasting up to 45 minutes and occurring without clear triggers. There were no recurrent visual or speech disturbances. While migraine and RCVS were considered, the latter was deemed less likely due to the absence of intracranial vascular abnormalities.

**Figure 5:**
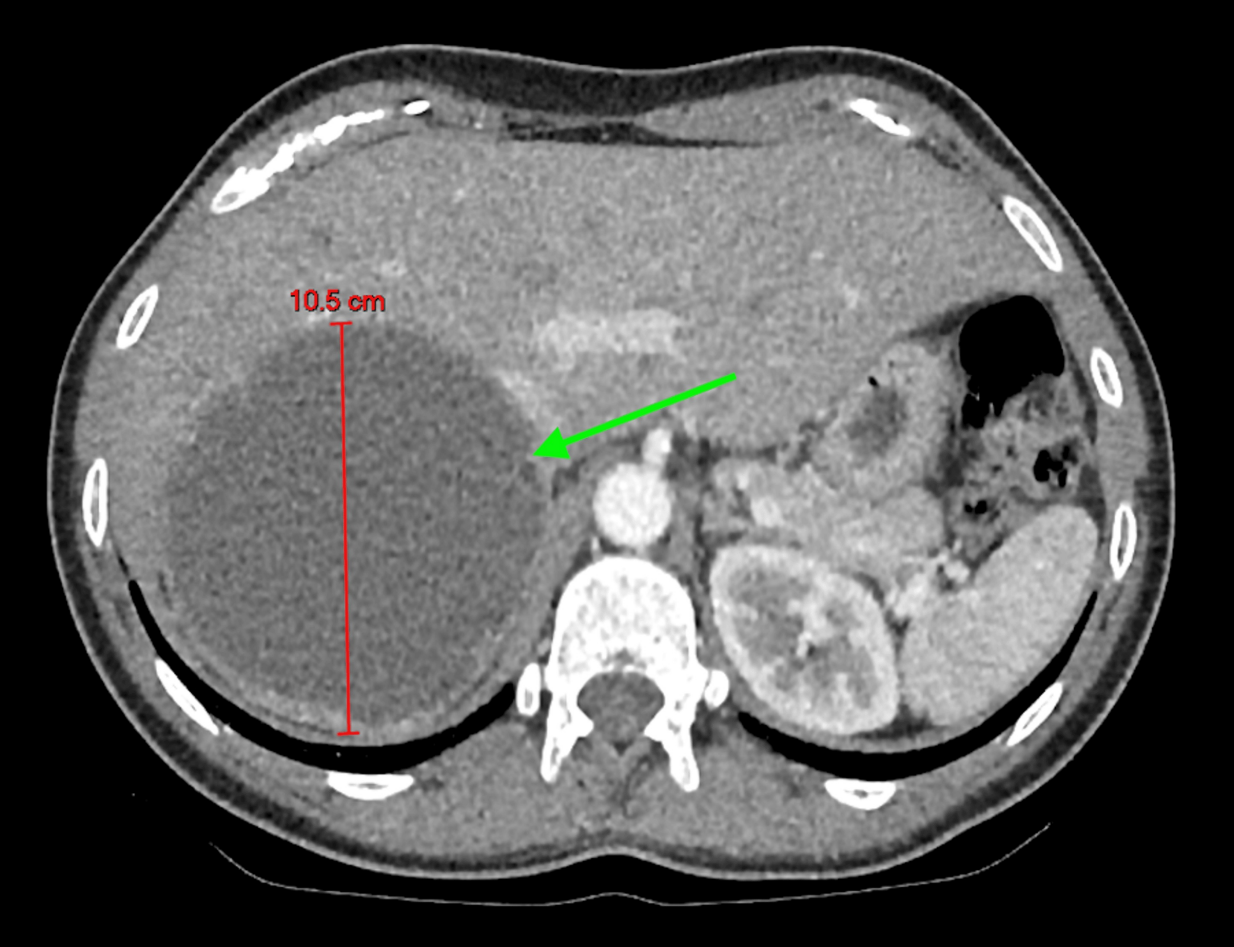
Contrast-enhanced CT of the abdomen revealing a 10.5 cm cystic mass in the right upper quadrant (green arrow), displacing the right kidney and indenting the liver.

She proceeded to have further evaluation in the private sector, where a bubble echocardiogram was done initially, which showed late bubbles in the left heart suggestive of intrapulmonary shunting. This was further investigated with a CT thorax to rule out pulmonary arteriovenous malformation, which incidentally showed a right upper quadrant mass (images of the CT of the thorax and echocardiogram are unavailable as the investigation was done in the private sector). Given the incidental findings, a CT abdomen was done, which identified a 10.5 cm cystic mass with Hounsfield units (HU) greater than 10 in the right upper quadrant (Figure 5), displacing the right kidney and indenting the liver. Endocrine workup (Table 1) revealed markedly elevated plasma normetanephrine and metanephrines, consistent with a catecholamine-secreting tumor. Cortisol, renin, and aldosterone levels were normal.

**Table 1:** Results of biochemical work up following results of the abdomen CT Lab results show elevated plasma normetanepherines and plasma metanepherines consistent with a catecholamine-secreting tumor. Plasma renin and aldosterone levels were normal. There was an appropriate response to cortisol following an overnight dexamethasone suppression test.

Laboratory Values	Reference Range (units)	Patient results
Plasma normetanephrine	<1180 (pmol/L)	11652 pmol/L
Plasma metanephrines	<510 (pmol/L)	5627 pmol/L
3-Methoxytyramine	<180 (pmol/L)	175 pmol/L
Renin concentration	<52 (mIU/L)	24.3 mIU/L
Cortisol (post overnight dexamethasone)	<50 (nmol/L)	33 nmol/L
Aldosterone	130-800 (pmol/L)	205 pmol/L

Treatment initiation was delayed as she was hospitalized in February 2024 with chest pain, later attributed to cocaine-induced coronary vasospasm with normal coronary vessels on CT coronary angiography. Alpha-blockade was initiated with doxazosin in March 2024, and beta-blockade was introduced one week before the elective laparoscopic adrenalectomy in May 2024 (Table 2).

**Table 2:** Alpha and beta blockade titration prior to adrenalectomy Doxazosin was used as an alpha blocker initially and started at a low dose of one milligram at night. The patient was reviewed weekly to ensure tolerance to medication, absence of postural blood pressure changes, and improvement in symptoms. The doxazosin was then titrated slowly to a maintenance dose of 2 mg twice daily prior to the addition of beta blockade with labetalol at 100 mg twice daily, one week prior to the scheduled adrenalectomy.

Day	Medication	Dose (mg/day)	Blood pressure (Sitting→ Standing)	Heart Rate (bpm)	Symptoms	Adjustments Made
1	Doxazosin	1mg at night	125/88→122/80	66	Intermittent Palpitations	Medication started
7	Doxazosin	1mg twice daily	106/79→108/85	65	Ongoing intermittent palpitations	Dose increased to twice daily
14	Doxazosin	1mg in the morning and 2 mg at night	108/83→107/80	70	Intermittent flushing palpitations	Dose increased to a total daily dose of 3mg
28	Doxazosin	2mg twice daily	104/75→103/70	66	Asymptomatic	Dose increased to 2mg twice daily
42	Doxazosin and Labetalol	Doxazosin 2mg twice daily and Labtelol 100mg twice daily	106/80→105/75	65	Asymptomatic	Doxazosin dose kept at maintenance. Labetalol was added one week prior to elective surgery.

**Figure 6:**
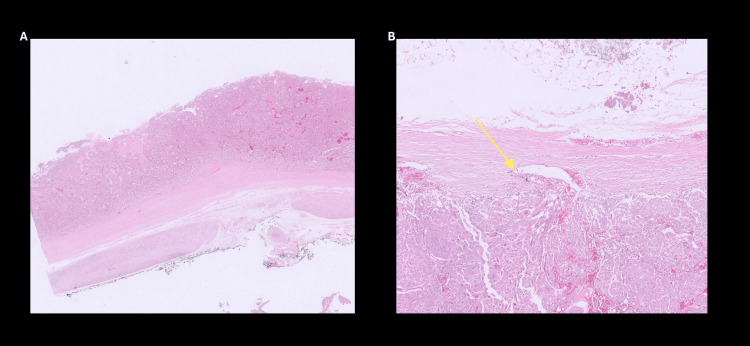
Histopathology slides showing: (A) an ultra-low-power hematoxylin and eosin (H&E) slide demonstrating an encapsulated neoplasm with central cystic degeneration and (B) a medium-power H&E image demonstrating lymphovascular space invasion (yellow arrow).

**Figure 7:**
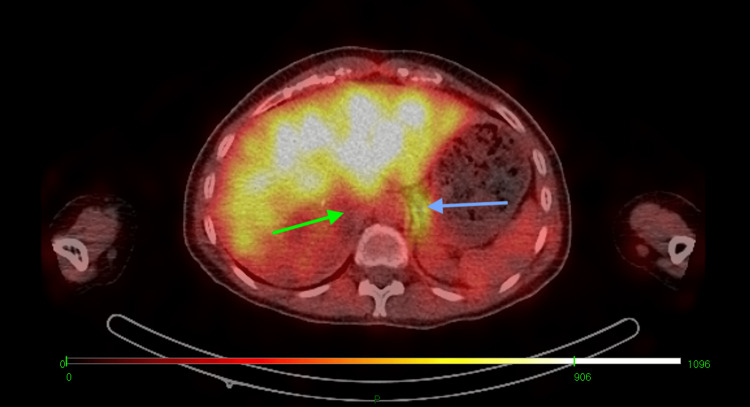
Postoperative metaiodobenzylguanidine (MIBG) scintigraphy showing absence of radiotracer uptake at the site of the resected right adrenal pheochromocytoma (green arrow), with normal physiological uptake in the left adrenal gland (blue arrow).

Histopathological examination confirmed a unifocal pheochromocytoma (pT2NxMx) with central cystic change (Figure 6A), confined to the adrenal capsule but demonstrating lymphovascular invasion (Figure 6B). The Ki-67 index was >3%. The Grading System for Adrenal Pheochromocytoma and Paraganglioma (GAPP) score was 7, and the Pheochromocytoma of the Adrenal Gland Scaled Score (PASS) was 10, collectively indicating a poorly differentiated tumor with a high metastatic potential. A metaiodobenzylguanidine (MIBG) scan performed three months postoperatively (Figure 7), as well as comprehensive genetic testing, was both unremarkable. Plasma metanephrines and normetanephrines had normalized at both three and six months postoperatively. In view of the normal biochemistry, negative MIBG findings, unremarkable genetic screening, and resolution of clinical symptoms, the multidisciplinary team recommended regular clinical surveillance without the need for further intervention.

## Discussion

This case illustrates a rare but clinically significant association between pheochromocytoma and RCVS, highlighting the diagnostic complexity posed by overlapping neurological and systemic symptoms. Although RCVS is increasingly recognized in association with catecholamine surges [3], cases secondary to pheochromocytoma remain exceedingly uncommon, with only limited examples reported in the literature in the last decade [6-8].

The patient’s initial presentation resembled a primary headache disorder, with normal early imaging and a lack of focal neurological signs, leading to a provisional diagnosis of migraine or cluster headache. Similar to our case, acute headaches secondary to RCVS have been previously diagnosed as migraine [3], and treatment with triptans can further exacerbate vasoconstriction [3]. The subsequent development of transient dysphasia is in keeping with literature that focal neurological deficits, commonly secondary to ischemic strokes [9], have been attributed to patients with RCVS [10]. 

This case presented unique features that set it apart from the more typical presentations of RCVS. Notably, the typical radiological findings and classical angiographic signs, such as the “string of beads” pattern, were absent. Initially, the vascular irregularities observed in the vertebral artery were interpreted as possible fibromuscular dysplasia or segmental arterial mediolysis, as these conditions can share similar clinical presentations and angiographic findings with RCVS. Although there are case reports and studies that suggest a potential association between RCVS and other vascular pathologies, the evidence to support these is limited [11-12].

Although RCVS is self-limiting and lasts up to three months, this patient experienced symptoms spanning more than a year, likely due to continued catecholamine excess from the undiagnosed pheochromocytoma. The other systemic manifestations expected in pheochromocytoma, including episodic palpitations, flushing, and vomiting, emerged only gradually. The eventual identification of a large adrenal mass during evaluation for suspected pulmonary shunting significantly altered the clinical trajectory. The complete resolution of symptoms and normalization of metanephrines following adrenalectomy strongly support the role of catecholamine surges from the pheochromocytoma in driving the patient’s cerebrovascular symptoms.

Despite these diagnostic ambiguities, the case offers valuable insights into the intersection of endocrine and neurovascular disorders. It highlights the importance of maintaining a broad differential diagnosis in patients presenting with recurrent thunderclap headaches, even when early imaging is unrevealing. This case contributes to the limited but growing body of literature describing pheochromocytoma-associated RCVS and reinforces the value of multidisciplinary collaboration in elucidating complex clinical presentations. Importantly, it demonstrates that the absence of typical imaging features should not preclude the diagnosis of RCVS when clinical suspicion remains high and systemic evaluation reveals a plausible precipitant. Greater awareness of this rare association may lead to earlier recognition and improved outcomes in similar cases.

## Conclusions

This case highlights an unusual and diagnostically challenging presentation of RCVS secondary to pheochromocytoma, marked by recurrent thunderclap headaches, stroke-like symptoms, and a prolonged, evolving clinical course. It underscores the limitations of early imaging, the potential for misdiagnosis, and the importance of considering endocrine causes in patients with atypical or persistent headache syndromes. The favorable clinical outcome following tumor resection emphasizes the reversibility of symptoms when the underlying cause is identified and treated. This case contributes to the current understanding of pheochromocytoma as a rare but important cause of secondary RCVS and supports the need for a systematic, multidisciplinary approach in similar complex presentations.

The clinical learning points are as follows: 1. RCVS may present without classical angiographic features and can mimic migraine or stroke, especially in the early phase. 2. Catecholamine-secreting tumors such as pheochromocytoma are a rare but important cause of secondary RCVS. 3. Early systemic evaluation and multidisciplinary input are crucial in patients with unexplained recurrent thunderclap headaches and evolving neurological or systemic symptoms.
